# Exercise Ameliorates Spinal Cord Injury by Changing DNA Methylation

**DOI:** 10.3390/cells10010143

**Published:** 2021-01-12

**Authors:** Ganchimeg Davaa, Jin Young Hong, Tae Uk Kim, Seong Jae Lee, Seo Young Kim, Kwonho Hong, Jung Keun Hyun

**Affiliations:** 1 Department of Nanobiomedical Science & BK21 FOUR NBM Global Research Center for Regenerative Medicine, Dankook University, Cheonan 31116, Korea; ganchimeg12@gmail.com (G.D.); vrt23@naver.com (J.Y.H.); 2 Institute of Tissue Regeneration Engineering (ITREN), Dankook University, Cheonan 31116, Korea; 3 Department of Rehabilitation Medicine, College of Medicine, Dankook University, Cheonan 31116, Korea; magnarbor@dankook.ac.kr (T.U.K.); rmlee@dankook.ac.kr (S.J.L.); juliet8383@naver.com (S.Y.K.); 4 Department of Stem Cell and Regenerative Biotechnology and Institute of Advanced Regenerative Science, Konkuk University, Seoul 05029, Korea; hongk@konkuk.ac.kr; 5 Wiregene, Co., Ltd., Cheonan 31116, Korea

**Keywords:** spinal cord injury, exercise, epigenetics, DNA methylation, 5-hydroxymethylcytosine, ten-eleven translocation

## Abstract

Exercise training is a traditional method to maximize remaining function in patients with spinal cord injury (SCI), but the exact mechanism by which exercise promotes recovery after SCI has not been identified; whether exercise truly has a beneficial effect on SCI also remains unclear. Previously, we showed that epigenetic changes in the brain motor cortex occur after SCI and that a treatment leading to epigenetic modulation effectively promotes functional recovery after SCI. We aimed to determine how exercise induces functional improvement in rats subjected to SCI and whether epigenetic changes are engaged in the effects of exercise. A spinal cord contusion model was established in rats, which were then subjected to treadmill exercise for 12 weeks. We found that the size of the lesion cavity and the number of macrophages were decreased more in the exercise group than in the control group after 12 weeks of injury. Immunofluorescence and DNA dot blot analysis revealed that levels of 5-methylcytosine (5mC) and 5-hydroxymethylcytosine (5hmC) in the brain motor cortex were increased after exercise. Accordingly, the expression of ten-eleven translocation (Tet) family members (Tet1, Tet2, and Tet3) in the brain motor cortex also elevated. However, no macrophage polarization was induced by exercise. Locomotor function, including Basso, Beattie, and Bresnahan (BBB) and ladder scores, also improved in the exercise group compared to the control group. We concluded that treadmill exercise facilitates functional recovery in rats with SCI, and mechanistically epigenetic changes in the brain motor cortex may contribute to exercise-induced improvements.

## 1. Introduction

Spinal cord injury (SCI) is a major devastating lesion, and neurological recovery after SCI is rarely seen in the clinical setting. Exercise training was recommended for SCI patients in the rehabilitation unit several decades ago. The main reason for locomotor training in SCI patients was initially to maximize the remaining muscle function to replace the impaired function caused by disconnected motor tracts in the spinal cord. However, some researchers have reported functional recovery after exercise training in animal SCI models, and they explained this phenomenon in three ways: compensation, regeneration, and neuronal plasticity. The compensation mechanism was explained by the proper balance among the central pattern generator below the lesion, sensory feedback, and the remaining descending pathways from the cerebrum and brainstem after SCI [1,2]. Regeneration of corticospinal tracts was enhanced by extracellular signal-regulated protein kinase 1/2 (ERK1/2) activation in SCI rats [3]. Numerous previous studies reported an increase in neuronal plasticity after locomotor training, with upregulation of various neurotrophic factors, including brain-derived neurotrophic factor (BDNF) and glial cell line-derived neurotrophic factor (GDNF) in the spinal cord [4,5] and BDNF, microtubule-associated protein 2 (MAP-2), and synaptophysin in the motor cortex [6], or modulation of the phosphatase and tensin homolog (PTEN)/mammalian target of rapamycin (mTOR) signaling pathway [7] in animal SCI models.

DNA methylation at the 5-position of cytosine (5mC) and demethylation through the conversion of 5mC to 5hmC is important to regulate axonal regeneration in the central and peripheral nervous systems [8]. Several epigenetic modifications have been reported promoting functional recovery following SCI [9,10]. Therefore, DNA methylation and demethylation may contribute to enhance regenerative capacity in SCI.

In our recent study, we showed that level of DNA demethylation is changed after SCI, and the administration of ascorbic acid could effectively promote DNA hypermethylation and enhance functional restoration in rat SCI models [11]. Previous studies have shown that metabolic and signaling changes during exercise may affect the epigenetic status of skeletal muscles [12,13,14] and the brain [15,16]; however, changes in epigenetic status caused by locomotor training have not yet been elucidated. Recently, hippocampal ten-eleven translocation 1 (Tet1) and Tet2 expression were found to increase after active exercise, which may be related to memory restoration in aged mice [17]. Our previous finding that Tet1 and Tet2 expression increased in the brain motor cortex was partially related to improvement in locomotor functions [11]; therefore, exercise-induced epigenetic changes may be expected to promote neuronal recovery after SCI.

The effects of exercise training on SCI have also been debated. Exercise following SCI is known to promote functional restoration and reduce neuropathic pain [18]. However, some researchers have reported that combination therapies using stem cell transplantation [19,20] and chemical agents [21,22] were effective for only functional improvements, and exercise even worsened allodynia via aberrant sprouting of C fibers through BDNF-tropomyosin receptor kinase B (TrkB) signaling [23] and exacerbated the inflammatory response after SCI [24].

In this study, we aimed to investigate whether treadmill exercise promotes histological and functional recovery in rats subjected to SCI and whether epigenetic changes are related to these results.

## 2. Materials and Methods

### 2.1. Animals and Surgical Procedures

**Figure 1:**
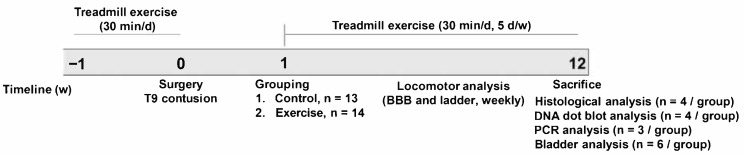
Timeline of the experiments.

Adult female Sprague–Dawley rats (12 weeks old, 230–250 g) were used in this study. All experimental procedures were approved by Dankook University’s Institutional Animal Care and Use Committee (approval No. 19-009). Animals were individually housed in clear plastic cages under a 12 h light/dark cycle with free access to food and water at room temperature (24 ± 1 °C) and a humidity of 45–50%. Rats were randomly divided into a control group without exercise (*n* = 13) and the experimental group, which received treadmill exercise (*n* = 14) (Figure 1)**.** The surgical procedures for SCI have been previously described in detail [25]. Briefly, animals were deeply anesthetized with isoflurane (Forane; Choongwae Pharma, Seoul, Korea), and T9 laminectomy was performed to expose the thoracic spinal cord. Moderate contusion injury (200 kdyn) was induced using the Infinite Horizon impactor (Precision Systems and Instrumentation, LLC, Fairfax, VA, USA). After SCI, the muscles and skin were sutured with sterile 5-0 sutures. Intramuscular injection of cefotiam hydrochloride (40 mg/kg; Fontiam™, Hanmi Pharma, Seoul, Korea) was performed in all operated rats for 3 days, and intraperitoneal injection of normal saline (0.9%; 3 mL) was performed just after surgery. Each rat also received oral administration of acetaminophen syrup (10 mg/kg; Tylenol™, Janssen Pharmaceutica, Titusville, NJ, USA) for 3 days, and manual bladder expression was performed twice daily until spontaneous bladder expression was reestablished.

### 2.2. Treadmill Exercise

Treadmill exercise training was performed using the Exer-3/6 open treadmill with shock for rats (Columbus Instruments, Columbus, OH, USA). The instrument supports exercising the rats in individual lanes at the same time. An electrical stimulus system option with shock grids was used for the exercise. The rats were exercised for 1 week before surgery for adaptation. The exercise schedule was started 1 week after injury, and treadmill exercise was performed twice per day (morning and afternoon for 15 min per session) and 5 days per week for 12 weeks after injury (Figure 1). Treadmill exercise was conducted at a running speed of 5 m/min at the beginning, and the speed was gradually increased to 12 m/min as the walking capabilities of the rats allowed until the end of the training as previously described [26,27].

### 2.3. Histology

Sections of the spinal cord and brain were analyzed by hematoxylin and eosin (H&E) and immunohistochemical staining. The rats were perfused with 0.9% normal saline, followed by 4% paraformaldehyde (Hushi Inc., Shanghai, China) in 0.1 M PBS (pH 7.4). The brains and spinal cords were dissected from the rats, postfixed overnight in 4% paraformaldehyde at 4 °C, and cryopreserved with 30% sucrose for 3 days. The tissue samples were embedded in the M1 compound (Thermo Fisher Scientific Inc., Waltham, MA, USA), the spinal cords were cut into 16 µm sagittal sections, and the brain samples were cut into 10 µm coronal sections using a cryotome cryostat. H&E staining was performed on the lesion epicenter to examine the general morphology of the spinal cord at 12 weeks after injury, and three sagittal sections containing a lesion cavity and four cases per group were evaluated for quantitative analysis. The size of the lesion cavity was manually outlined for each section under confocal microscopy and analyzed using Image J software (1.37 v, National Institutes of Health, Bethesda, MD, USA) as our previous studies [28,29].

### 2.4. Immunohistochemistry (IHC)

Immunohistochemistry was used to analyze DNA methylation within the brain after treadmill exercise and to observe the inflammatory status in the contused spinal cord. Sections were permeabilized with 0.2% Triton X-100 in 1X phosphate-buffered solution (PBS) for 5 min, washed with 1X PBS, and blocked with 10% normal goat serum in 1X PBS for 1 h. The primary antibodies, mouse anti-5-methylcytosine (5mC) (1:500, Active Motif, Carlsbad, CA, USA), rabbit anti-5-hydroxymethylcytosine (5hmC) (1:500, Active Motif), mouse anti-NeuN (1:100, Millipore, Bedford, MA, USA), rabbit anti-NeuN (1:1000, Abcam, Cambridge, MA, USA), guinea pig anti-NeuN (1:500, Synaptic Systems, Goettingen, Germany), mouse anti-GFAP (1:1000, MilliporeSigma, St. Louis, MO, USA), rabbit anti-GFAP (1:1000, DAKO Cytomation, Carpinteria, CA, USA), mouse anti-monocyte/macrophage ED1 (1:400, Millipore), rabbit anti-5HT (1:2000, MilliporeSigma), rabbit anti-Tet1 (1:100, Abcam), rabbit anti-Tet2 (1:100, Millipore) and rabbit anti-Tet3 (1:100, Santa Cruz, Inc., CA, USA), were incubated overnight at 4 °C. The sections were washed three times and treated with secondary antibodies. The secondary antibodies included FITC-, Alexa 594-, and Alexa 647-conjugated antibodies for monoclonal and polyclonal primary antibodies (1:200, Jackson ImmunoResearch, West Grove, PA, USA). After 2 h of incubation at room temperature, the sections were washed three times with PBS, and the cell nuclei were stained with DAPI (1:1000, MilliporeSigma). The stained tissue sections were imaged using confocal microscopy (Carl Zeiss Inc., Oberkochen, Germany).

Our analysis specifically focused on DNA methylation and demethylation in mature neurons in layers IV-V of the primary motor cortex (M1) within the brain after spinal cord injury. For quantification of the 5mC and 5hmC fluorescence intensity, three representative images of the primary motor cortex (M1) of each case and four cases per group were captured at 200X magnification on confocal microscopy. The average intensity of 5mC or 5hmC co-staining with NeuN was measured using ImageJ software (National Institutes of Health). For quantitation of ED1-positive monocytes and macrophages in the sagittal section, three images per case and four cases per group were captured at the lesion site using a confocal microscope and then counting the expressed cell numbers within a lesion cavity manually. The mean values of images were expressed as ED1-positive cell numbers per 1 mm^2^ in each case. Additionally, we examined the intensity of TET family proteins in the captured images. All images were captured using identical microscopy acquisition settings.

For quantification of 5HT axons, three images at the lesion cavity per case and four cases per group were selected. A threshold value was constant for every image, and the density of 5HT-positive axons was expressed. The number of positive pixels was then divided by the total number of pixels using Image J software as previously described [11,30].

We performed NF200 diaminobenzidine (DAB) staining, the sections were washed with PBS, and the peroxidase activity was blocked with 0.3% H_2_O_2_ in DW for 30 min at room temperature. After rinsing, the sections were incubated in 0.2% Triton-X in PBS for 5 min, 2% NGS in PBS for 1 h at room temperature, and then in the primary rabbit anti-NF200 (1:100 Millipore) antibody in 2% NGS at 4 °C overnight. Then, the sections were incubated in biotinylated goat anti-rabbit secondary antibody (1:200 Jackson ImmunoResearch) in 2% NGS for 2 h at room temperature, followed by incubation in the Vectastain Elite ABC elite kit (Vector Laboratories, Burlingame, CA, USA) for 30 min. Finally, NF200 staining was revealed with DAB solution (0.05% 3–3-diaminobenzidine tetrahydrochloride (MilliporeSigma), 0.06% NiCl2 (MilliporeSigma), and 0.003% H_2_O_2_), and the reaction was stopped with distilled water. The sections were dehydrated, coverslipped and allowed to dry. The lesion cavity was outlined manually from captured images, and the number of pixels occupied by the NF200-labeled axons from three images per case and four cases per group within the outlined lesion cavity was quantified using ImageJ software (National Institutes of Health). NF200-labeled axonal density was determined by dividing the number of NF200-positive pixels by the number of pixels in a field as described in our previous study [29].

Macrophage subsets in the contused spinal cord were analyzed using immunohistochemistry. The primary antibodies, rabbit anti-GFAP (1:1000, DAKO Cytomation), rabbit anti-monocyte/macrophage ED1 (1:400, Abcam), mouse anti-arginase I (1:50, Santa Cruz) and mouse anti-CD86 (1:100, BD Pharmingen, San Diego, CA, USA), were incubated overnight at 4 °C. After the sections were washed three times, goat anti-rabbit (Alexa Fluor 546, Jackson ImmunoResearch) and goat anti-mouse (Alexa Fluor 488, Jackson ImmunoResearch) secondary antibodies were used at a dilution of 1:200 in 2% NGS in PBS. Following 2 h of incubation, the sections were washed three times with PBS. The stained tissue sections were imaged using confocal microscopy (Carl Zeiss Inc., Oberkochen, Germany).

Macrophage polarization was quantified by dividing the number of CD86- or arginase I-positive cells by the number of ED1-positive cells with manual cell counting. The images were captured at the lesion site using a confocal microscope to assess the percentages of CD86-positive M1 and Arg1-positive M2 macrophages as previously described [29].

### 2.5. DNA Dot Blot Assay

Genomic DNA was extracted from the brain motor cortex in the control and exercise groups using the DNeasy blood and tissue kit (Qiagen, Hilden, Germany) to determine the amount of 5mC and 5hmC. Purified genomic DNA samples were spotted on a nitrocellulose membrane (0.2 µm pore size) using a 96-well manifold apparatus (Manifold I; Schleicher and Schuell, Dassel, Germany). DNA was immobilized to the membrane by baking at 80 °C for 2 h. The membrane was then blocked with 5% skim milk and incubated with mouse anti-dsDNA (1:2000, Abcam), rabbit anti-5mC (1:1000, Active Motif), or rabbit anti-5hmC (1:2000, active motif) at room temperature (RT) for 1 h. The membrane was incubated with a horseradish peroxidase-conjugated antibody specific for mouse or rabbit (Jackson ImmunoResearch) for 1 h at RT and visualized by enhanced chemiluminescence (Thermo Fisher Scientific Inc.). The relative amounts of 5hmC and 5mC in genomic DNA were calculated using ImageQuant TL software (GE Healthcare, Chicago, IL, USA).

### 2.6. Real-Time PCR

To examine the global changes in the expression of the DNA methylation-related genes and inflammation- and regeneration-related genes, the relative mRNA levels of Tet family members (Tet1, Tet2, Tet3), DNA methyltransferase (DNMT) 1, DNMT3a, tumor necrosis alpha (TNFα), interleukin (IL)-6, IL-1β, Krüppel-like factor-4 (Klf4), insulin-like growth factor-1 (IGF-1), and IL-10 in the brain motor cortex tissues were evaluated using real-time PCR. Briefly, total RNA was extracted using an RNeasy mini kit (Qiagen). cDNA was synthesized from 2 mg of total RNA with random hexamer primers and SuperScript III (Invitrogen, Thermo Fisher Scientific Inc.). Primers were designed with the UCSC Genome Bioinformatics and the NCBI database and are listed in Table 1. qRT–PCR was conducted with Fast SYBR green master mix (Applied Biosystems, Thermo Fisher Scientific Inc.) on a StepOne real-time PCR system (Applied Biosystems). All experiments were carried out in triplicate, and the expression of each gene was normalized to endogenous GAPDH mRNA and calculated as the relative fold change compared to the control groups.

### 2.7. Functional Assessments

We used two methods for assessments of the locomotor recovery of paralyzed hindlimbs after SCI, namely, the Basso, Beattie, and Bresnahan (BBB) locomotor rating scale and the horizontal ladder walk test (*n* = 13 in the control group and *n* = 14 in the exercise group). The BBB scale ranges from 0 points (no hindlimb movement) to 21 points (normal hindlimb movement) [31]. Two examiners observed hindlimb movements for 4 min in the open field (cylindrical-shaped acrylic box, 90 cm in diameter and 15 cm high, with a smooth floor). In the horizontal ladder test, the rats were trained several times for adaptation before testing. The rats run across a 127 cm long horizontal runway made from 3 mm diameter metal rungs with a minimum distance of 1 cm and two 10 cm tall acrylic sidewalls. The test was video recorded and analyzed in slow motion using a digital camcorder. The ladder score was presented as the percentage of erroneous steps among the total hindlimb steps [32]. The locomotor function of each group was examined every 7 days until the date of sacrifice.

Evaluations of bladder function and urodynamic studies (UDS) were performed according to previous studies [25,33,34]. Briefly, the rats were anesthetized with isoflurane, and an incision was made to expose the bladder. A 4-Fr double-lumen polyethylene catheter was used to connect the bladder to a pressure transducer, and an infusion pump (KDS100; KD Scientific Inc., Holliston, MA, USA) loaded with a normal saline-filled syringe. The pressure signal was amplified and recorded using LabChart software (PowerLab 8/30 with LabChart Pro; AD Instruments, Colorado Springs, CO, USA), and the measured parameters were the maximal micturition pressure (cmH_2_O) and frequency (time/min), the gross voiding patterns, and the regularity of the micturition pressure and frequency. Immediately before UDS, the diameter of the exposed bladder dome was measured for indirect evaluation of bladder hypertrophy. The bladder volume was calculated as an imaginary sphere as follows: bladder volume = (horizontal diameter of the bladder/2)^2^ × vertical diameter of the bladder/2 × π × 4/3.

### 2.8. Statistics

All numerical data are reported as the means ± standard deviations, and IBM SPSS Statistics 25 (IBM, Armonk, NY, USA) was used for analysis. The Shapiro–Wilk test was performed to check the normal distribution of all quantified histological and functional data from each group, and according to the results, a nonparametric test was chosen. For histological, immunohistochemical, DNA dot blot, and quantitative PCR data, the Mann–Whitney U test was performed to compare data between the control and exercise groups. Repeated measures two-way analysis of variance was used to compare locomotor function for 12 weeks, including the BBB and ladder tests, between the control and exercise groups followed by Bonferroni test for comparison between two groups at each time point. Effects were considered significant at *p* < 0.05

## 3. Results

### 3.1. Treadmill Exercise Reduces the Lesion Cavity and Inflammatory Response after SCI

**Figure 2:**
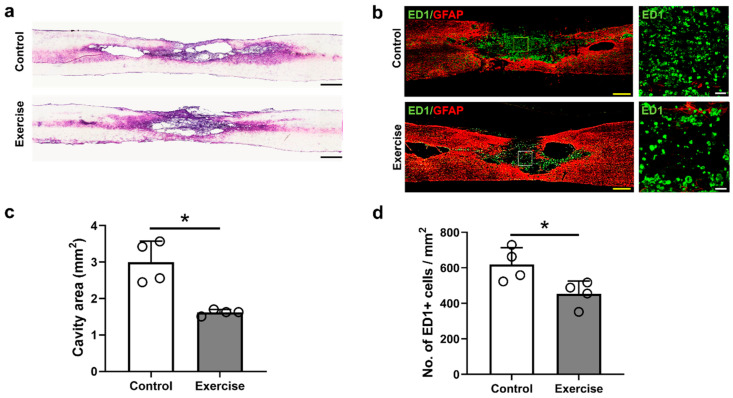
Histological analysis of the injured spinal cords at 12 weeks after injury. Representative images of (**a**) H&E staining and (**b**) immunofluorescent staining for ED1 (green) and GFAP (red) in the control and exercise groups. (**c**) The cavity area measured from the sagittal images of H&E staining (*n* = 4 per group). (**d**) Number of ED1-positive macrophages counted in the lesion area from immunohistochemical staining (*n* = 4 per group). * *p* < 0.05 between the control and exercise groups by the Mann–Whitney U test. Black and yellow scale bars = 500 μm, white scale bar = 100 μm.

We measured the size of lesion cavities via H&E staining of injured spinal cord tissue and found that the cavity size of the control group without exercise was larger than that of the experimental group, which performed exercise until the time of sacrifice (3.00 ± 0.58 mm^2^ and 1.62 ± 0.08 mm^2,^ respectively, Figure 2a,c). The number of ED1-positive cells within the lesioned epicenter was also higher in the control group one in the 12 weeks exercise group after injury (1429.0 ± 395.6 and 735.8 ± 140.2, respectively, Figure 2b,d).

### 3.2. Treadmill Exercise Modulates DNA Methylation and Hydroxymethylation in the Brain Motor Cortex after SCI

**Figure 3:**
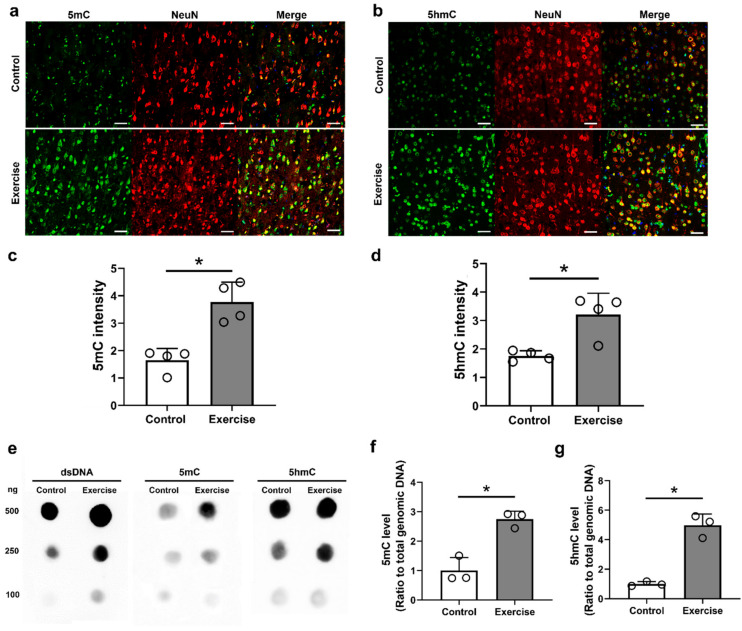
Epigenetic changes in the brain at 12 weeks after injury. Representative immunofluorescent images of double staining of (**a**) 5mC (green) and NeuN (red) and (**b**) 5hmC (green) and NeuN (red) in the primary motor cortex of the control and exercise groups. (**c**) 5mC and (**d**) 5hmC intensity in neurons in the primary motor cortex of the control and exercise groups (*n* = 4 per group). (**e**) Representative dot blot results for the primary motor cortex with 100, 250, and 500 ng of 5mC and 5hmC in the control and exercise groups. Quantitative data from dot-blot analysis of 250 ng of (**f**) 5mC and (**g**) 5hmC compared with the amount in total genomic DNA in the control and exercise groups. * *p* < 0.05 between the control and exercise groups by the Mann–Whitney U test. White scale bars = 50 µm.

We investigated DNA methylation changes in the brain motor cortex at 12 weeks after injury in both the control and exercise groups. 5mC and 5hmC within neurons in the brain motor cortex were identified using immunohistochemical double-staining (anti-NeuN and anti-5mC antibodies, or anti-NeuN and anti-5hmC antibodies) (Figure 3a,b), and we found that the anti-5mC and anti-5hmC intensities within NeuN-positive neurons were higher in the exercise group than those in the control group (1.65 ± 0.43 and 3.77 ± 0.72 for anti-5mC intensity, and 1.76 ± 0.18 and 3.21 ± 0.75 for anti-5hmC intensity, respectively, Figure 3c,d).

Additionally, the amounts of 5mC and 5hmC in genomic DNA extracted from the brain motor cortex were analyzed using DNA dot blot assay (Figure 3e). We found that the 5mC and 5hmC levels in 250 ng of genomic DNA were significantly higher in the exercise group than those in the control group (2.75 ± 0.27 for 5mC and 4.97 ± 0.76 for 5hmC in the exercise group, Figure 3f).

**Figure 4:**
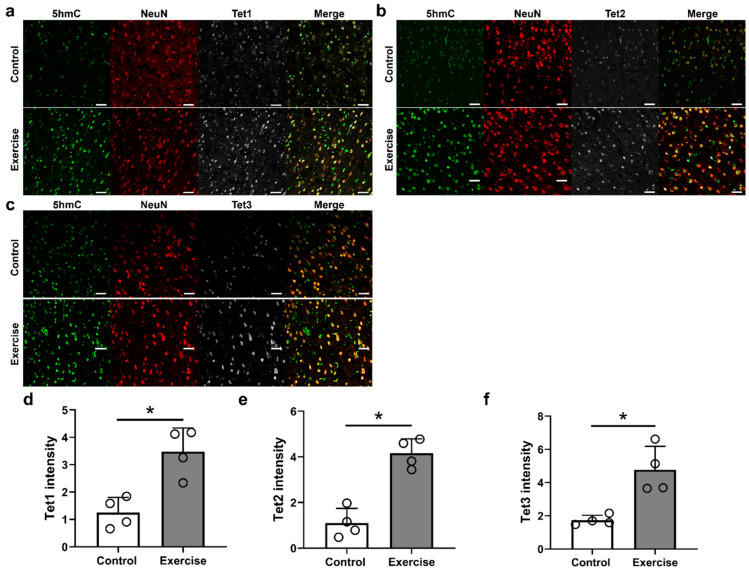
Representative immunofluorescent images showing triple staining of 5hmC (green), NeuN (red) and (**a**) Tet1 (gray), (**b**) Tet2 (gray), or (**c**) Tet3 (gray) in the primary motor cortex 12 weeks after injury in the control and exercise groups. (**d**) Tet1, (**e**) Tet2 and (**f**) Tet3 intensity of 5hmC-stained neurons in the primary motor cortexes of rats in the control and exercise groups (*n* = 4 per group). * *p* < 0.05 between the control and exercise groups by Mann–Whitney U test. White scale bars = 50 µm.

Tet enzymes catalyze the conversion of 5mC to 5hmC, and further conversion of 5hmC to 5-formylcytosine (5fC) and then to 5-carboxycytosine (5caC). We, therefore, identified whether the increase in 5hmC after exercise training was associated with the Tets. Neurons in the brain motor cortex were triple-stained with anti-5hmC, anti-NeuN, and one of three different anti-Tet isoforms (TET1, 2, and 3) antibodies (Figure 4a–c. We found that the anti-Tet1, anti-Tet2, and anti-Tet3 intensities of the exercise group were significantly higher than those of the control group (1.25 ± 0.56 and 3.47 ± 0.87 for anti-Tet1 intensity, 1.10 ± 0.64 and 4.16 ± 0.64 for anti-Tet2 intensity, 1.73 ± 0.30 and 4.77 ± 1.41 for anti-Tet3 intensity, respectively, Figure 4d–f.

### 3.3. Treadmill Exercise Promotes Axonal Regeneration and Sprouting, but Do Not Affect Macrophage Polarization in the Lesion Cavity after SCI

**Figure 5:**
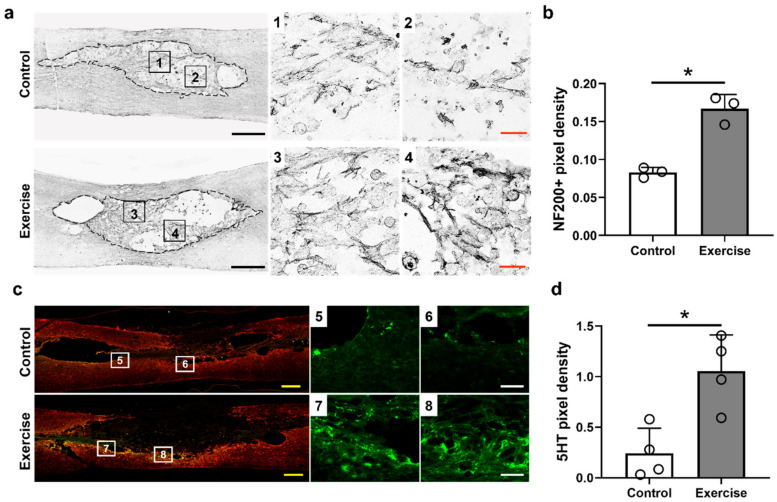
NF200 DAB staining results (**a**,**b**) and 5HT immunohistochemical results (**c**,**d**) in the injured spinal cord tissue at 12 weeks after injury (**a**–**d**). (**a**) Representative images of NF200-positive axons in the control and exercise groups. The right images are high-magnification images of the black boxes in the left images (Nos. 1–4) within the lesion cavities of contused spinal cords (outlined by black dashed lines). (**b**) The density of NF200-positive axons of the control and exercise groups (*n* = 4 per group). (**c**) Representative images of 5HT-positive axons (green) and GFAP-positive astrocytes (red) in the control and exercise groups. The right images are high-magnification images of the black boxes in the left images (Nos. 5–8) adjacent to the lesion cavities of contused spinal cords. (**d**) The density of 5HT-positive axons of the control and exercise groups (*n* = 4 per group). * *p* < 0.05 between the control and exercise groups by Mann–Whitney U test. Black and yellow scale bars = 500 μm, white scale bar = 100 μm, red scale bar = 50 μm.

We found that NF200-positive axons within the lesion cavity were increased in the exercise group more than in the control group (Figure 5a), and the density of NF200-positive axons was also higher in the exercise group than in the control group (0.083 ± 0.006 for the control group and 0.167 ± 0.018 for the exercise group, Figure 5b). We also found that 5HT-positive axons were increased more around the lesion cavity in the exercise group than in the control group (0.24 ± 0.25 for the control group and 1.06 ± 0.36 for the exercise group, Figure 5c,d). These findings suggest treadmill exercise may enhance axonal regeneration and sprouting in SCI models.

**Figure 6:**
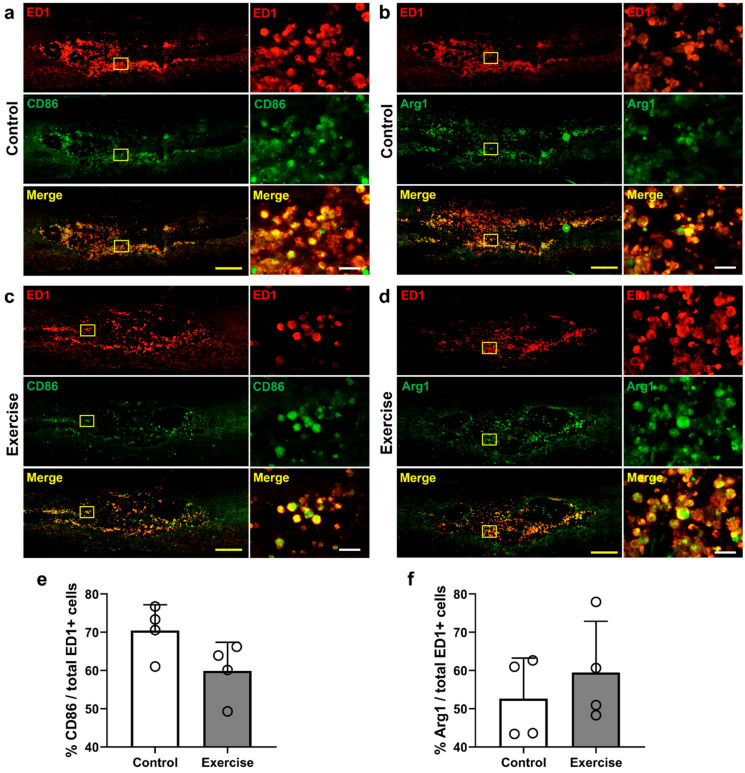
Immunofluorescent findings related to macrophage M1/M2 polarization 12 weeks after injury. (**a**) Representative images of CD68- and ED1-positive M1 macrophages and (**b**) Arg1- and ED1-positive M2 macrophages in the control group and (**c**) CD68- and ED1-positive M1 macrophages and (**d**) Arg1- and ED1-positive M2 macrophages in the exercise group. (**e**) Number of cells positive for (**e**) CD86 and (**f**) Arg1, normalized to ED1-positive cells (*n* = 4 per group). *p* < 0.05 between the control and exercise groups by the Mann–Whitney U test. Yellow scale bar = 500 μm, white scale bar = 50 μm.

We performed double-staining with anti-CD86 and anti-ED1 antibodies for the detection of M1 macrophages, and anti-Arg1 and anti-ED1 antibodies for the detection of M2 macrophages in injured spinal cord tissue at 12 weeks after injury (Figure 6a–d. The overall numbers of ED1-positive macrophages were smaller in the exercise group, as previously described; however, the proportion of CD86- and Arg1-positive macrophages to total (ED1-positive) macrophages was not significantly different between the control and exercise groups (Figure 6e,f).

**Figure 7:**
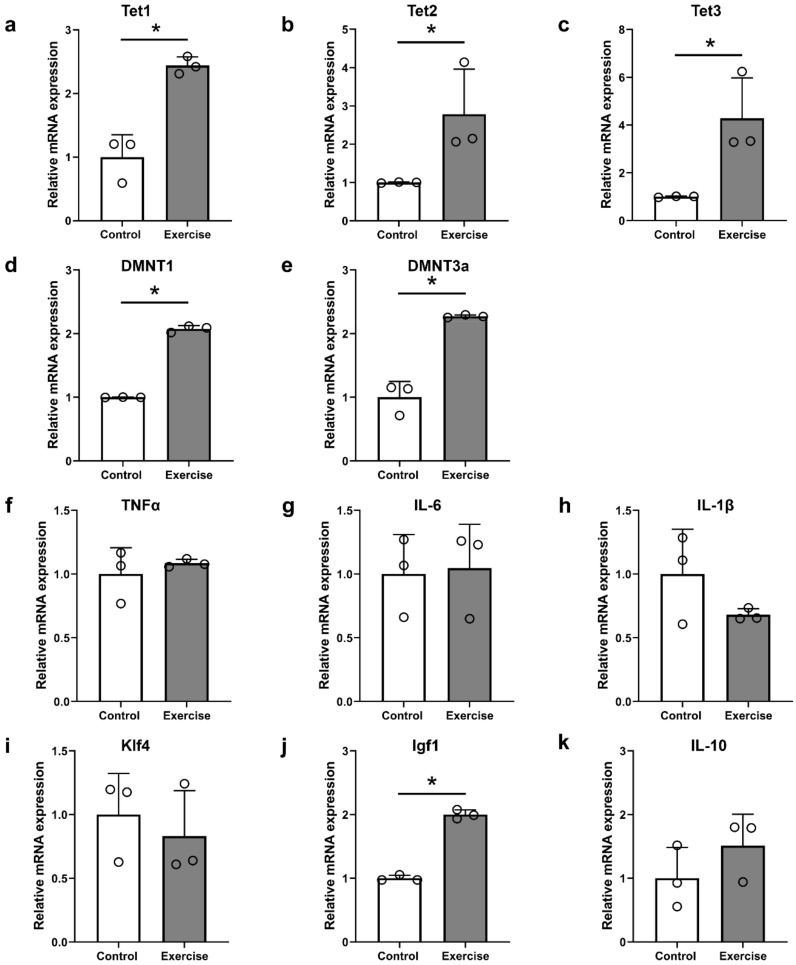
Real-time PCR analysis in the primary motor cortex of the control and exercise groups at 12 weeks after injury. (**a**–**c**) Tet family genes (Tet1, Tet2, and Tet3), (**d**,**e**) DNA methyltransferase genes (DNMT1, DNMT3a), (**f**–**h**) inflammation-related genes (TNFα, IL-6, and IL-1β), and (**i**–**k**) regeneration-related genes (Klf4, Igf1, IL-10) (*n* = 3 per group). * *p* < 0.05 between the control and exercise groups by the Mann–Whitney U test.

We analyzed the changes in gene expression after exercise in SCI models using qRT–PCR analysis (Figure 7). The expression levels of Tet1, Tet2, and Tet3 were all increased in the exercise group compared to the control group (Figure 7a–c, and the expression levels of different DNA methyltransferase genes (DNMT1a and DNMT3) were also increased in the exercise group (Figure 7d,e). The expression levels of inflammation-related genes (TNFα, IL-6, and IL-1β) were not different between the control and exercise groups (Figure 7f–h, and one of the regeneration-related genes, Igf1, was increased in the exercise group (Figure 7j), whereas Klf4 and IL-10 were not different between the control and exercise groups (Figure 7i,k).

### 3.4. Treadmill Exercise Promotes Functional Improvement, but Do Not Modulate Neurogenic Bladder after SCI

**Figure 8:**
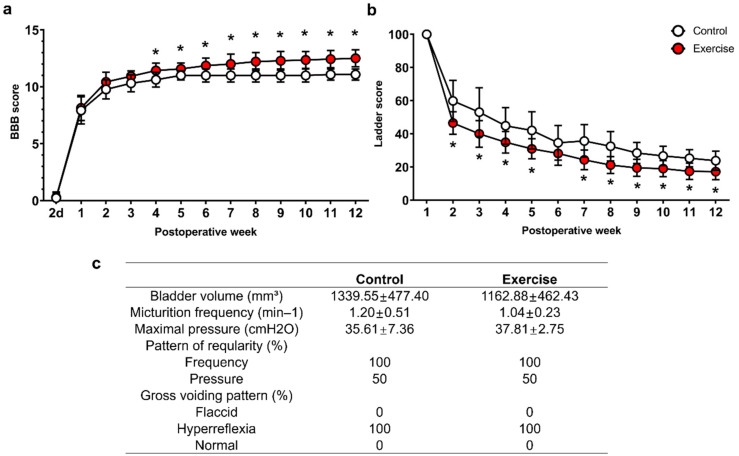
Functional recovery of spinal cord-contused rats in the control and exercise groups. The locomotor functions of rats with SCI were analyzed using (**a**) Basso, Beattie, and Bresnahan (BBB) scores and (**b**) horizontal ladder tests during the 12-week experimental period (*n* = 13 for the control group, and *n* = 14 for the exercise group). (**c**) The results of the urodynamic study and measurement of bladder volume at 12 weeks after injury (*n* = 6 per group). * *p* < 0.05 between the control and exercise groups by repeated-measures two-way ANOVA followed by Bonferroni test at each time point.

Locomotor function was assessed by measuring BBB and ladder scores. The BBB score was higher in the exercise group than in the control group from 4 weeks after injury to the time of sacrifice, and the final scores at 12 weeks after injury were 11.1 ± 0.5 and 12.5 ± 0.8 in the control and exercise groups, respectively (Figure 8a). The ladder score showed a better recovery pattern in the exercise group from 2 weeks after injury to the time of sacrifice, and the final scores at 12 weeks after injury were 23.8 ± 5.8 and 17.2 ± 4.8, respectively, in the control and exercise groups (Figure 8b). The bladder volume, and bladder functions, which were evaluated using urodynamic study, showed typical hyperreflexic pattern; increased frequency and maximal pressure 12 weeks after SCI (Figure 8c), and the exercise group showed similar bladder volume and hyperreflexic voiding pattern without any improvement (Figure 8c).

## 4. Discussion

We found that genes and pathways associated with DNA methylation and hydroxymethylation, including 5mC, 5hmC, Tet1, Tet2, Tet3, DNMT1, and DNMT3a, were activated in the brain motor cortex after treadmill exercise during the 12-week period following SCI (Figure 3, Figure 4 and Figure 7). DNMT1 and DNMT3a are members of the family of DNA methyltransferase enzymes that convert cytosine to 5mC, and 5mC is hydroxylated into 5hmC by Tet1, Tet2, and Tet3 [35,36]. Therefore, in our study, treadmill exercise promoted the methylation and hydroxymethylation of cytosine in the brain motor cortex.

The roles of 5mC and 5hmC in the hippocampus were partially uncovered [17,37]. Jessop et al. found that Tet1 and Tet2 expressions in the hippocampus were decreased in aged mice, and voluntary exercise for five weeks induced increases in Tet1 and Tet2 expression and 5hmC levels; memory function was also improved [17]. In addition, DNA methylation may increase gene expression related to neuronal plasticity and improve memory and cognitive function in the hippocampus [37]. Enhanced DNA methylation, increased 5mC levels, and DNMT1 activity induced by maternal supplementation with folic acid were also shown to improve the early development of motor and sensory functions in rat offspring [38]. Previously we showed that 5mC levels in genomic DNA were increased in the acute and subacute stages; 5hmC levels in genomic DNA, and mRNA expression levels of Tet1, Tet2, and Tet3 were also increased from the acute to the chronic stage in the brain motor cortex after SCI [11]. Therefore, increased DNA methylation and hydroxymethylation in the brain motor cortex may provide a chance to enhance the neuronal plasticity of damaged corticospinal tracts in the lesioned spinal cord.

Axonal regeneration after SCI is thought to be closely related to changes in DNA methylation and hydroxymethylation, and these changes appear differently depending on the type of gene in the spinal cord [39]. The brain motor cortex is the location of the cell body of the corticospinal tracts and is also important for controlling hindlimb activity during locomotion in cats and rats [40,41]. The motor cortex also influences motor recovery following neuromodulation [42] and reorganizes substantially following task-specific tasking after SCI [43].

Epigenetic changes in the brain motor cortex following exercise after SCI were not evident in a previous study; however, several notable results have been found in skeletal muscles and the brain in previous studies [12,13,14,15,16]. The gene expression changes that occur following exercise are not clearly regulated by DNA methylation, but relative DNA hypomethylation of key metabolic and regulatory genes in skeletal muscle may be associated with exercise training [12]. Although the global change of DNA methylation has been examined in other studies, the results are different according to the type and duration of exercise training, and since DNA methylation or demethylation is related to individual gene changes, it is necessary to determine the changes for each gene [13,14]. Moreover, various epigenetic changes are already known to occur after trauma or disease in the central nervous system [44], and it is very difficult to predict additional epigenetic changes after exercise. Our previous study revealed that global DNA methylation and hydroxymethylation in the brain motor cortex were increased according to the duration after SCI, and ascorbic acid enhanced 5mC and 5hmC formation within the brain motor cortex at three months after application, with a concomitant functional improvement [11]. Sun et al. also found that 5hmC was increased in spinal cord tissue during the acute stage after SCI, and inhibition of Tet2 exacerbated necrosis within the lesioned spinal cord, although no epigenetic changes within the brain were reported [45].

Studies have reported the effectiveness of exercise training in spinal cord injury in animal models, and humans, especially for the functional restoration and control of neuropathic pain [46,47,48,49], and that axonal sprouting and synaptic plasticity were increased after treadmill exercise in SCI rodents [47,50]. DNA methylation is also important for facilitating synaptic plasticity [51], and inhibition of DNA methylation induced the impairment of long-term hippocampal potentiation in rats in a previous study [52]. We found that NF200-positive axons exhibited more outgrowth within the lesion cavity (Figure 5a,b), and 5HT-positive axons, which might represent axonal sprouting after SCI, were visible more around the lesion cavity (Figure 5c,d) in the spinal cord in exercise-treated rats than in the control rats although synaptic plasticity was not investigated in this study. In addition to axonal regeneration, axonal sprouting caudal to the injured spinal cord may be related to functional improvement in SCI [53], and some treatments that induce 5HT axonal sprouting have successfully promoted functional recovery after SCI in previous studies [54,55]. As shown in the results in this study, locomotor functions, which were evaluated using BBB and ladder scores, were also improved during the 12-week experimental period (Figure 8).

Bladder function is frequently disrupted in SCI patients, and neurogenic bladder causes urinary tract infection, incontinence, urinary stones, and poor quality of life. The most frequent bladder dysfunctions observed in thoracic SCI patients are detrusor hyperreflexia and detrusor-sphincter dyssynergia [56], and thoracic spinal cord-contused rats also showed the same pattern in our previous studies [33,34]. Previously, forelimb exercise training was shown to reduce maximal pressure during bladder contraction using awake cystometry, and whole-body vibration was effective in minimizing incontinence [57]. In our previous study using sham-operated controls, bladder volume was 564.54 ± 134.01 mm^3^, micturition frequency was 0.60 ± 0.21/min, maximal pressure was 26.81 ± 5.81 cmH_2_O, and gross voiding pattern was normal [33]. Therefore, when compared with the values of the previous study, bladder volume, voiding frequency and maximal pressure, and detrusor hyperreflexia after SCI were not recovered in both the control and exercise groups (Figure 8b); therefore, we could not find any improvements in bladder dysfunction in the exercise group. More than three-quarters of all SCI patients cannot void volitionally [58], and the recovery of bladder function is more complex than that of hindlimb locomotion, as it involves somatic and autonomic pathways that are disrupted or deactivated after SCI. Further study of bladder recovery after SCI is needed to obtain an effective treatment.

Previous studies have revealed macrophage changes after exercise in SCI [46,59]. Chhaya et al. found that the number of macrophages in the dorsal root ganglion below the lesioned spinal cord was reduced early after 4-week exercise training, and neuropathic pain was also improved [46]. However, in another study, the number of microglia in the lumbar spinal cord after thoracic spinal cord contusion was not decreased, although the locomotor function was improved early after two weeks of treadmill exercise [59]. In this study, we found that numbers of macrophages and microglia in the lesioned spinal cord decreased in number after treadmill exercise (Figure 2b,d), while the ratio of M1/M2 macrophages and the levels of M2 macrophage-related IL-10 were not changed (Figure 6, Figure 7k). Some treatments modulating M2 polarization of macrophages and microglia have also shown functional restoration after SCI [29,60]; however, there has been no study revealing the relationship between M2 polarization and exercise after SCI, and the epigenetic mechanisms regulating M1/M2 polarization have not yet been determined [8].

Compared to a previous study using ascorbic acid, 100 mg/kg ascorbic acid and 11-week treadmill exercise showed similar patterns, with an increase in global DNA methylation, and some proinflammatory cytokines, such as IL-1β and TNFα, were not changed in the brain motor cortex 12 weeks after SCI [11]. At the chronic stage after SCI, proinflammatory cytokines are usually downregulated, but our previous study showed the optimal concentration of ascorbic acid was effective in decreasing IL-6 [11], which was not found in this study. However, in order to clarify the effect of treadmill exercise on the proinflammatory cytokines as well as macrophage polarization, further study is necessary at the acute stage where the inflammatory process is maximum. Some regeneration-associated genes, including Klf4 and Igf1, showed opposite patterns of expression, and DNMT1 and DNMT3a gene expression after exercise was increased, whereas it was not changed after ascorbic acid application [11]. The Igf1 gene is a regeneration-associated gene that shows a neuroprotective effect against apoptosis, and treadmill exercise after SCI promoted the upregulation of Igf1 gene expression in previous studies [27,61], whereas Klf4 gene expression was downregulated following treadmill exercise [27]. Igf1 was effective in regrowth of corticospinal tracts after the application of osteopontin to spinal cord-injured mice in a previous study [62], and the upregulation of Igf1 expression after exercise may have contributed to axonal outgrowth in the corticospinal tract and functional restoration in this study. Klf4 gene is a reprogramming factor, and its overexpression combined with Oct4 overexpression improved BBB scores in SCI mice [63]; however, some researchers reported opposite findings. For example, Klf4-suppressed retinal ganglion cells showed an increase in axonal outgrowth [64], downregulation of Klf4 reduced the inflammatory response and enhanced functional outcomes in SCI rats [65], and deletion of Klf4 promoted axonal regeneration of the corticospinal tract in SCI mice [66]. Changes in the Igf1 and Klf4 genes after the application of ascorbic acid or treadmill exercise training may not be related to epigenetic changes, and further research is needed to reveal their relationships with functional improvements. The upregulation of DNMT1 and DNMT3a expression within the brain motor cortex is related to increased 5mC intensity and an increased amount of 5mC in genomic DNA in the 12 weeks exercise group after SCI. In our previous study, 5mC intensity was not increased after ascorbic acid application in the chronic stage [11]; therefore, treadmill exercise is more sustainably effective for DNA methylation than ascorbic acid. It is unclear whether these results are related to the persistence of the functional recovery effect after SCI, but it seems clear that our results show patterns of epigenetic changes that differ from those associated with ascorbic acid application. Therefore, combinations of drugs and exercise, which can be applied in the clinical setting for SCI patients, may have a synergistic effect in promoting functional restoration in SCI, and further studies will be planned. In addition, since several mechanisms including ERK1/2 activation, upregulation of various neurotrophic factors, and compensation mechanisms may contribute the effect of treadmill exercise on functional restoration after SCI, the effect of DNA methylation and demethylation in this study could not be identified separately from these mechanisms. Therefore, efforts to confirm the effect of epigenetic changes on functional restoration after exercise through additional experiments that inhibit DNA methylation or demethylation will be required.

## 5. Conclusions

In this study, we found that DNA hypermethylation was enhanced after treadmill exercise in the SCI rats, and treadmill exercise also facilitated functional recovery. We concluded that epigenetic changes in the brain motor cortex might contribute to exercise-induced functional improvements.

## Figures and Tables

**Table 1:** Primer sequences used for real-time PCR gene expression analysis.

Gene	Forward (5′-3′)	Reverse
Tet1	GGCTTGCAGACACTGATGAA	GAAACACAGTCGCCTCTTCC
Tet2	GGGGTTGGAGCAAGTACAAA	CGGGTGTGTGTCATTTGAAG
Tet3	AGTGGGTGATCCGAAGACAC	GCCAGGATCAAGATGACGAT
DNMT1	GTGTGCGGGAATGTGCTCGCT	CAGTGGTGGTGGCACAGCGT
DNMT3a	AGCAAAGTGAGGACCATTACCACCA	TGTGTAGTGGACAGGGAAGCCA
TNF-α	CTCAAGCCCTGGTATGAGCC	GGCTGGGTAGAGAACGGATG
IL-6	ACCACCCACAACAGACCAGT	CAGAATTGCCATTGCACAAC
IL-1β	CACCTCTCAAGCAGAGCACAG	GGGTTCCATGGTGAAGTCAAC
Klf4	CTTTCCTGCCAGACCAGATG	GGTTTCTCGCCTGTGTGAGT
Igf1	CACACTGACATGCCCAAGAC	GGGAGGCTCCTCCTACATTC
IL-10	CAGCTGCGACGCTGTCATCG	GCAGTCCAGTAGATGCCGGGT
18 s rRNA	CACTGAGCATCTCCCTCACA	GAGGGTGCAGCGAACTTTAT

## Data Availability

The data presented in this study are available on request from the corresponding author.
